# Epidemiological Characteristics of Patients Operated for Achilles Tendon Rupture in Shanghai

**DOI:** 10.1111/os.13347

**Published:** 2022-06-22

**Authors:** Zhao‐lin Teng, Sheng‐xuan Cao, Xin Ma, Xu Wang, Jia‐Zhang Huang, Chao Zhang, Xiang Geng

**Affiliations:** ^1^ Department of Orthopedic Surgery, Huashan Hospital Fudan University Shanghai China

**Keywords:** Achilles tendon rupture, Epidemiology, Shanghai, Sports, Warm‐up

## Abstract

**Objective:**

To reflect the potential epidemiological characteristics of Achilles tendon (AT) rupture in Shanghai, China, which has been rarely reported before.

**Methods:**

This work is a descriptive epidemiology study. A total of 302 cases of AT rupture admitted to our department between 01/2013 and 02/2020 are analyzed according to telephone follow‐up and medical records. Male to female ratio is 10.3 and the average age is 37.5 years. The record of each case includes age, gender, Body mass index (BMI), quinolone use, corticoid exposure and related medical history/comorbidities. If the case is sports‐related (SR), details including kind of sports, intensity of exercise, exercise time before rupture, specific action that causes rupture and situation of warm‐up are collected. Two independent sample *t*‐tests and Pearson chi‐square tests are used for statistical analysis.

**Results:**

A total of 252 ruptures are SR. Male to female ratio is 15.6 in SR cases. Most SR ruptures occur in patients aged 25–39 years. Ball games are major sports responsible for rupture: basketball in 95 (37.7%), badminton in 68 (27.0%) and soccer in 62 (24.6%). Acceleration and running start is the specific action that cause most (37.7%) ruptures. AT cases are observed in 91 patients with warm‐up and 161 without preparation before exercise. As a result, more ruptures happened within 10 min' sports in 161 unprepared (22.4%) than in 91 prepared (5.5%) cases. In SR cases, 107 and 145 cases are observed on weekends and weekdays. Of the 302 total cases, 64 are associated with Achilles tendinopathy. Frequently reported factors such as quinolone use and corticoid exposure are found only in two and 11 of all cases, respectively.

**Conclusion:**

Middle‐aged males are common victims of AT rupture in Shanghai. Sports including basketball, badminton, soccer and actions involving in sudden and severe contraction of AT cause most ruptures. Warm‐up before exercise reduces rupture in short time. Factors such as quinolone, corticoid and Achilles tendonitis still need attention.

## Graphical abstract

The study aims to discover the epidemiology of Achilles tendon rupture in Shanghai, China. Basic information, causes, sports details and relevant factors are investigated to analyze the epidemiological characteristics.

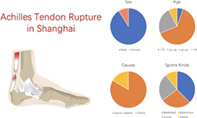

## Introduction

Achilles tendon (AT) rupture is a common disease in the orthopedic department. In recent years, the incidence of AT rupture keeps increasing worldwide.^1^, ^2^, ^3^, ^4^, ^5^, ^6^ People who actively participate in sports such as soccer players and basketball players are exposed to a great danger of AT rupture.^7^, ^8^, ^9^ AT is the most powerful tendon in the human body, mainly controlling the ankle activity. Upon rupture, ankle plantarflexion is severely weakened. Mobility recovery after surgery or conservative treatment often takes over half a year for patients. Unsatisfactory prognosis which usually is limited range of activities and fear of rupture often turn into obstacles for sports.

The epidemiological research of AT rupture has been carried out in the US,^10^, ^11^, ^12^ Canada,^13^ New Zealand^14^ and many developed countries in Europe.^1^, ^2^, ^3^, ^4^, ^5^, ^6^ In most reports, the vast majority of rupture are related to sports. Males make up more cases due to their active participation in sports. The ruptures are distributed more amongst middle‐aged than other age groups. Many studies conclude that middle‐aged men who occasionally take part in vigorous sports at weekends have a great risk of AT rupture, for which they are called “weekend warrior”.^7^, ^10^, ^15^, ^16^ Sports related to rupture vary greatly in different countries: basketball, tennis, and football in America,^10^ soccer and volleyball in Europe^1^, ^2^, ^3^ and Canada,^13^ and netball in New Zealand.^14^ Differences in related sports reflect preferences and uniqueness of national sports and lead to the various incidences of AT rupture in different countries and areas. Moreover, BMI, quinolones and corticoids are reported frequently in AT rupture.^17^, ^18^, ^19^, ^20^, ^21^, ^22^, ^23^ High or normal BMI may increase the risk of AT rupture according to previous research, but the opinion remains controversial.^17^ It has been proved that local or systematic use of quinolones and corticoids will do damage to AT and contribute to the rupture.^17^, ^18^, ^19^, ^20^, ^21^, ^22^, ^23^ The current results suggest not using quinolones and corticoids simultaneously for the amplified harm.^21^, ^22^, ^23^ Some other factors like seasonal variation is reported as well: the sport‐related AT ruptures happened most in spring whereas non‐sport‐related ruptures were distributed evenly throughout the year.^24^

Research on the epidemiology of AT rupture in China is limited. The sports related to AT rupture are seldom studied. Though the actions involving violent contractions of AT are considered the most dangerous triggers theoretically, it is not specific without actual data. Whether warm‐up helps reduce AT rupture or not is unclear so far. This paper reflects the potential characteristics of AT rupture in Shanghai by investigating a group of patients diagnosed with AT rupture. Considering habits and sports preferences, this paper hypothesizes that the main characteristics of AT rupture in Shanghai differ from those in European and American countries. The aims of this paper are: (i) to help analyze the high‐risk population; (ii) to discuss common risk factors of AT rupture; and (iii) to discover the role of sports and give advice on avoiding rupture.

## Methods and Materials

### Inclusion and Exclusion Criteria

This epidemiology study describes cases of AT rupture included according to the following criteria.

The inclusion criteria are as follows: (i) patients were admitted to our department and received surgery because of AT rupture; and (ii) diagnoses were proven by discontinuity of AT in surgery.

The exclusion criteria are as follows: information of the patient cannot be reconfirmed and collected by telephone follow‐up.

Our department is one of the centers of foot and ankle surgery in the area. By searching the medical record database between January 2013 and February 2020, 376 cases were listed. To assure the accuracy and completeness of the records, the patients in the list were contacted by telephone instead of directly using medical records. Finally, 302 cases were followed up. The remaining cases were not analyzed.

### Data Processing

#### Follow‐up Records

The records of each patient include age, gender, BMI, occupation, kind of sports, exercise intensity (recreational, lower competitive or top level^25^), exercise time before rupture, warm‐up, action that caused rupture, rupture time, occupation, amount of exercise, related diseases and history, tobacco or alcohol use, quinolone use and corticoid exposure.

#### Grouping Details

The cases related to sports were defined as sports‐related (SR) cases and those caused by other factors were denoted as other‐related (OR) cases.

As to age, patients were divided to four groups, namely, Young: ≤24, Middle age: 25–39, Middle elderly: 40–54, Old:≥ 55, as shown in Table 1.

**TABLE 1:** Basic information in SR/OR

	Age				BMI					Gender	
	Young	Middle age	Middle elderly	Old	Thin	Normal	Overweight	Obese	Severe obese	Male	Female
SR	14	178	54	6	3	93	101	41	14	237	15
OR	2	14	17	17	0	16	20	12	2	38	12
Total	16	192	71	23	3	109	121	53	16	275	27

Comparison of number of SR and OR cases as stratified by Age and BMI of the patients. Age: Young ≤24, Middle age 25–39, Middle elderly 40–54, Old ≥55

BMI: Thin <18.5, Normal 18.5–23.9, Overweight 24–26.9, Obese 27–29.9), Severe obese ≥30. SR, sports‐related; OR, others‐related

According to the Chinese standard of BMI, the patients were sorted into five groups, namely, Thin (<18.5 kg/m^2^), Normal (18.5–23.9 kg/m^2^), Overweight (24–26.9 kg/m^2^), Obese (27–29.9 kg/m^2^) and Severe obese (≥30 kg/m^2^), as shown in Table 1.

According to the exercise time before the rupture, patients were divided into Short (≤10 min), Middle (11–59 min), Long (≥ 60 min) and Unknown groups, as shown in Table 2.

**TABLE 2:** Warm‐up, medical history and exercise time before rupture

	Exercise time				
	Short	Middle	Long	Unknown	Total
Warm‐up					
With	5	43	42	1	91
Without	36	57	58	10	161
Medical history					
With	6	19	17	2	44
Without	35	81	83	9	208
Total	41	100	100	11	252

Comparison of SR ruptures happening in specific exercise time, between people who do warm‐up and do not, and between people with medical history and without.

Exercise time: Short (≤10 min), Middle (11–59 min), Long (≥ 60 min). SR, sports‐related.

#### Ankle Activity Score

The Ankle Activity Score^11^ lists many sports, activities and work. Exercise is divided to three levels: recreational, lower competitive or top level. Top level includes international elite, professional, national team and first division. Recreational level means participating in the sports just for physical activity without any scoring competition. Lower competitive level is between these two levels such as amateur competition. According to the exercise level, each kind of activity gets a score range from 0 to 10. 10 means the most intense and 0 mean disabled to move. The score quantifies the intensity of exercise. Using the Ankle Activity Score,^25^ the amount of exercise was calculated by intensity × time, and classified into five groups, namely, Group I—patient is more active on weekends; Group II—patient is as active on weekdays as on weekends; Group III—patient is less active on weekends; Group IV—patient does no sports or physical work; and Unknown Group, as shown in Table 3.

**TABLE 3:** Rupture time and amount of exercise

	Amount of exercise				
	I	II	III	IV	Unknown
Rupture time					
Weekends	45	26	21	12	3
Weekdays	41	43	28	30	3
Total	86	69	49	42	6

Comparison of rupture occurred on weekends and on weekdays as grouped by amount of exercise

Group I: Patient is more active on weekends; Group II: Patient is as active on weekdays as on weekends; Group III: Patient is less active on weekends; Group IV: Patient do no sports or physical work; and Unknown Group

### Statistical Analysis

Statistical analysis was performed by SPSS version 23.0 (IBM, Armonk, NY, USA). When comparing ages between males and females, two independent sample *t*‐tests were operated. Pearson chi‐square tests were used for other intergroup comparisons. A *P* value less than 0.05 (*α* = 0.05) was defined as significant.

## Results

### Basic Information

#### Gender

In the 302 total cases, 275 ruptures occurred in 267 males (three bilateral, five reoperation), whereas 27 ruptures occurred in 26 females (one reoperation), resulting in a male to female ratio of 10.3. In 252 SR cases, 237 ruptures occurred in 234 males (two bilateral, one reoperation), whereas 15 ruptures were observed in females, increasing the ratio to 15.6.

#### Age

The age at time of rupture ranges from 16 to 73, with an average age of 37.5 years (median age, 36 years). The average age of males is 37.0 years old, younger than females, 43.0 years old (*P* = 0.043). In SR cases, the largest group is Middle age, accounting for 178 (70.6%), as shown in Table 1. In OR cases, the groups Middle elderly and Old each have 17 cases. For patients younger than 40 years old, sports plays a more important role in ruptures than others (SR 92.3% *vs* OR 7.7%). In the older patients (Middle elderly and Old), OR cases increased to 36.2%, although SR cases were still the majority (63.8%).

#### *BMI*

The BMI data are shown in Table 1. The average BMI is 25.0 (range, 17.1–33.1). No significant difference was observed between SR and OR cases as to BMI composition ratio (*p* = 0.647).

### Etiological Details

#### Causes

For SR cases, the sports what patients were involved in were reviewed (Table 4). Basketball, badminton and soccer are the most common kinds that caused AT rupture. Others such as volleyball, rope skipping, tennis, running, yoga and football account only 27 cases. For the 50 OR cases, 12 (24.0%) were walking as usual when AT broke, and 12 (24.0%) stepped on empty steps. Other causes include trauma and falling down.

**TABLE 4:** Sports and action before rupture

Sport		Actions causing ruptures	
Basketball	95 (37.7)	Acceleration	91 (36.1)
Badminton	68 (27.0)	Take‐off of Jump	44 (17.5)
Soccer	62 (24.6)	Landing of Jump	41 (16.3)
Running	7 (2.8)	Rapid Turn	30 (11.9)
Rope Skipping	6 (2.4)	Rapid Stop	10 (4.0)
Volleyball	2 (0.8)	Back step	7 (2.8)
American Football	2 (0.8)	Constant Running	8 (3.2)
Other sports	10 (4.0)	Others	11 (4.4)

The number of ruptures related with each kind of Sport and Action. Number in parentheses is percentage of SR cases. SR, sports‐related

#### Actions

The specific actions that led to ruptures in 252 SR cases were investigated (Table 4). Though 10 patients did not remember the situation, the remaining 242 described their action in detail, which were summarized as eight kinds: Acceleration (including Running Start), Take‐off of Jump, Landing of Jump, Rapid Turn, Rapid Stop, Back Step, Constant Running and Others. The first four actions all involve a great load on AT, accounting for 81.7% of SR cases.

#### Warm‐Up

In 252 SR cases, 91 (36.1%) got prepared well by warming up whereas 161 (63.9%) did not prepare before sports activities (Table 2). The composition of exercise time before rupture is not the same between prepared and unprepared patients (*P* < 0.001). Ruptures happened more in 10 min in 161 patients who skipped warm‐up (36, 22.4%) than those in 91 prepared (5, 5.5%). After 10 min of exercise (including Middle and Long Groups), no significant difference was observed in exercise time between prepared and unprepared patients (*P* = 0.886).

### Daily Exercise

#### Rupture Time

In all 302 cases, 124 ruptures occurred on weekends (Saturday and Sunday), namely 107 in 252 SR cases and 17 in 50 OR cases. In SR cases, 107 and 145 occurred on weekends and weekdays, respectively. Compared with OR cases, more SR cases occurred on weekends (42.5% *vs* 34.0%) although not significant (*P* = 0.267).

#### Exercise Baseline

By interviewing the patients regarding their daily activities, the amount of exercise was recorded in the form of “sport kind × time” relatively for weekdays and weekends. If one does SR work or physical work that is defined as active work according to the Ankle Activity Score, his/her work was recorded as well to be calculated as part of the amount of exercise. The rupture happened more on weekends than on weekdays only in Group I (Table 3).

#### Occupation

The occupations were divided into two kinds: active work, as mentioned before, and static work such as office worker, programmer, student and retiree. A total of 232 (76.8%) out of the 302 cases and 200 (79.4%) out of the 252 SR cases engaged in static work.

### Medical History of Achilles Tendon

Twenty OR cases and 44 SR cases were recorded with a medical history of Achilles tendinitis, AT pain and AT rupture/tear (ipsilateral or contralateral) before the rupture and/or with degeneration found in operation. No difference was observed between patients with and without related medical history as to exercise time before rupture (Table 2, *P* = 0.938) or sports kind (Table 5, *P* = 0.985).

**TABLE 5:** Relation between medical history and sports kinds

	Sport			
	Basketball	Badminton	Football	Others
Medical history				
With	17	12	11	4
Without	78	56	51	23
Total	95	68	62	27

### Rupture Side

In terms of rupture side, 100 cases were right and 152 were left in 252 SR cases. The idiomatic foot of patients were inquired in the follow‐up (Table 6). Most patients had to right foot injuries (208 *vs* 42) as expected. AT rupture of one side clearly happened more in people who habitually used the same side (*P* = 0.011).

**TABLE 6:** Effect of idiomatic side on rupture

	Idiomatic side			
	Left	Right	Both	Total
Rupture side				
Left	34	117	1	152
Right	8	91	1	100
Total	42	208	2	252

### Other Factors

Medicine such as quinolones or corticoids were acknowledged as risk factors of AT rupture. In 302 cases, only two patients (0.7%) used quinolones within 3 months before rupture, six patients (2.0%) received local corticoids injection and five (1.7%) were exposed to oral/intravenous corticoids. No one used quinolones and corticoids at the same time. Other factors such as the comorbidity of diabetes was reported in four patients (1.3%), and use of euthyrox was reported in two patients (0.7%). In all cases, 97 (32.1%) and 103 (34.1%) patients were recorded with smoking and alcohol use, respectively. Two patients wore unsuitable shoes when rupture occurred, that is, one's shoes were tight and the other's shoes were loose.

## Discussion

### Main Findings of the Study

A male to female ratio of 10.3 is shown in AT ruptures. Sports‐related ruptures happen most in Middle age (25–39). Basketball, badminton and soccer are the most common sports that caused AT rupture. Acceleration, Take‐off of Jump and Landing of Jump take up 70.0% of actions before rupture. Warm‐up reduces rupture happened in 10 min' exercise from 22.4% to 5.5%. More SR cases occurred on weekends than OR cases (42.5% *vs* 34.0%). Only 21.2% cases were recorded with relevant diseases before rupture.

### Basic Information

According to the census in 2010, the local gender ratio (male to female) is 1.0619.^26^ In this paper, the ratio is 10.3 overall and 15.6 in SR cases. Moreover, females are 6 years older than males when rupture happened. The finding is similar to European,^1^, ^2^, ^3^ Canadian^13^ and American studies,^10^ and even gender ratio is bigger. However, they are quite different from the New Zealand study.^14^ Strenuous exercise such as basketball and soccer are the most common incentives, which are not prior choices to women in this area. In the New Zealand study, females' participation in netball resulted in a unique result that the ratio is 1.0 and females are younger.

The distribution of AT rupture in different age groups was evaluated as well. In the census in 2010,^26^ the proportions of these four age groups are 24.8%, 28.8%, 23.8% and 22.6%. In SR cases, Middle age takes up 70.6%. The Young and Old groups take up only 5.6% and 2.4% in AT rupture compared with the census, respectively. People younger than 24 have better physical conditions and stronger self‐healing ability, whereas people older than 55 take minimal part in strenuous exercise due to traditional living habits.

The risk of high BMI on AT rupture has been studied recently.^17^ To integrate with the actual situation better, the sorting standard was based on Chinese demographic data. The BMI data in our paper accorded with a normal distribution with the average BMI 25.0. Over half of the patients were overweight and even obese, but no significant difference was noted amongst BMI in OR cases, BMI in SR cases and local demographic BMI data,^27^ which was consistent with Noback's study,^17^ indicating that BMI is not a risk factor. Another study believed that normal BMI increases the risk of AT disorders.^20^ The effect of BMI on the recovery of AT rupture remains controversial. BMI was thought to influence the outcomes of surgery, which was supported by Chido.^18^ However, a recent study showed that obesity does not affect ankle function and pain relief after surgery of AT rupture.^19^

### Etiological Details

Sports involvement is evident in AT rupture. A total of 83.4% cases were associated with various sports in this paper, more than other studies.^10^, ^14^ In different countries, the involved sports are different from one another. Basketball, badminton and soccer are the most three sports causing ruptures, while basketball, tennis, and football in America,^10^ soccer and volleyball in Europe^1^, ^2^, ^3^ and Canada,^13^ and netball in New Zealand.^14^ From the data of each year alone, the top three causes are always the three sports, with small changes in the ranking. The results show that in recent years, the popularity of sports remains unchanged. Considering the importance of ball games in rupture (accounting for more than 89.3% cases) and gender of ball players, understanding the high gender ratio of 15.6 in this paper is easy.

Specific actions were reported for the first time to study the mechanism in SR cases. The most common action is Acceleration (36%), followed by Take‐off of Jump, Landing of Jump and Rapid Turn. The contraction of AT and great AT load is similar in these four actions. The frequency of actions may contribute to the disparity. Previous studies have shown that microdamage, chronic inflammation in local microenvironment and reduction of vessels cause the damage and incomplete repair of AT.^16^, ^28^, ^29^ Our paper proves that the sudden, severe contraction of AT, especially when the foot is at maximum dorsiflexion, is most commonly the last straw.

Warm‐up activities are acknowledged as a way to reduce SR injuries. Indeed, fewer prepared patients after warm‐up were exposed to rupture in a short time(≤10 min) of exercise. However, after 10 min of exercise, which may play the same role as warm‐up, the exercise time before rupture was approximately the same. Our result cannot prove that warm‐up does help to reduce AT rupture because of the absence of a control group. A further control study can work on the actual benefits of warm‐up on AT rupture.

### Daily Exercise

A total of 107 and 145 of SR cases occurred on weekends and weekdays, respectively, whereas 17 and 33 of OR cases occurred on weekends and weekdays, respectively. Considering only two of seven days are weekends, SR cases seems to be more likely to occur on weekends than on weekdays, and OR cases are distributed evenly. To study the phenomenon further, Table 3 reveals that for people who are more active on weekends (Group I), AT rupture also happens more on weekends. For Group IV, rupture happens evenly. The unexpected result is that in Groups II and III, ruptures on weekends did not decrease apparently. The small sample size may partly explain the result. Another reason is that several patients did the same sports on weekends and on weekdays, and the exercise time was equal or less but more concentrated on weekends than on weekdays. The intensity of sports on weekends was often more severe as well.

Briefly, males between 25 and 39, who engaged in static work and concentrate on ball games in a specific short time (often on weekends) or exercise occasionally have a large risk of AT rupture. The finding is similar to the “weekend warrior” mentioned before in many studies.^7^, ^10^, ^15^, ^16^

### Medical History of AT

Only 44 patients were diagnosed with AT diseases, and over 82.5% of patients did not feel uncomfortable or get alarmed before the rupture. One reason explaining for this is many people pay much attention to avoid rupture and even leave sports once they are diagnosed with AT diseases or feel uncomfortable. Moreover, AT rupture is somehow unexpected and unpredictable, reminding sports fans to take care of their strongest tendon.

### Rupture Side

One belief holds that for people who are right handed, their left AT is more likely to rupture.^16^ Our result is that the left AT is more vulnerable, that is, no matter if the individual is left/right footed, patients lose more left AT than right AT (60.3% *vs* 39.7%). The influence of idiomatic foot is also reflected: right‐footed individuals experience more right rupture than the overall (43.8% *vs* 39.7%), and left‐footed individuals experience more left rupture than the overall (81.0% *vs* 60.3%). In conclusion, for left‐footed individuals, the left AT is more likely to rupture; for right‐footed individuals, both sides are dangerous, and the left tends to rupture to a slightly greater extent. The habit and regularity of dangerous actions in sports and the long‐term damage of the idiomatic foot are the possible reasons.

### Other Factors

Quinolone or corticoid use was minimal in our paper because of the changing usage habits of antibiotics and steroids as well as the awareness of risk factors. Considering that most patients are male and their living habits, the high use of cigarettes (32.1%) and alcohol (34.1%) is understandable. Whether the use of cigarettes and alcohol harms AT and causes rupture needs more research.

Two patients described improper use of shoes in AT rupture, one's shoes were tight and the other's shoes were loose. Recent studies have shown the effects of shoes on AT load,^30^, ^31^ indicating the importance of wearing suitable shoes to prevent rupture.

### Limitations

Limitations include small sample size and data source. The sample excluded those patients who received conservative treatment instead of operation. All data came from only one hospital, which created geographical and social bias. The medical record contents of patients varied from 2013 to 2020, and several records were incomplete. By telephone follow‐up, the contents were integrated, which decreased credibility especially for cases which happened several years ago. Connecting with databases in other hospitals with a customized record of AT rupture is the better way to study the characteristics of AT rupture.

### Conclusion

In Shanghai, most AT ruptures occur during exercise. Middle‐aged men who prefer ball games are exposed to a great risk of AT rupture. Basketball, badminton and soccer are the most popular but dangerous sports leading to rupture. Actions related to the sudden, severe contraction of AT such as Acceleration and Running Start are the most common direct causes of rupture. Sufficient warm‐up may help reduce the possibility of rupture. BMI is not a risk factor for AT rupture, but a high BMI may delay the recovery of AT. The left side is vulnerable to AT rupture, especially in left‐idiomatic patients, which contradicts previous studies. Before rupture, only a few patients have related medical history or complaints, indicating that an omen is often ignored, and AT rupture is difficult to prevent. Other factors such as quinolone or corticoid use have a minimal effect because of improved medical knowledge. For people who engage in sports, wearing suitable shoes is always important because to prevent discomfort and AT rupture.

## Funding

This work was supported by grants from Shanghai Shen Kang Hospital Development Center (SHDC2020CR3071B). The sponsors or funders had no involvement in any part of this study. All authors confirmed the independence of researchers from funding sources.
